# Schizophrenia-associated mt-DNA SNPs exhibit highly variable haplogroup affiliation and nuclear ancestry: Bi-genomic dependence raises major concerns for link to disease

**DOI:** 10.1371/journal.pone.0208828

**Published:** 2018-12-10

**Authors:** Christian M. Hagen, Vanessa F. Gonçalves, Paula L. Hedley, Jonas Bybjerg-Grauholm, Marie Bækvad-Hansen, Christine S. Hansen, Jørgen K. Kanters, Jimmi Nielsen, Ole Mors, Alfonso B. Demur, Thomas D. Als, Merete Nordentoft, Anders Børglum, Preben B. Mortensen, James Kennedy, Thomas M. Werge, David M. Hougaard, Michael Christiansen

**Affiliations:** 1 Department for Congenital Disorders, Statens Serum Institut, Copenhagen, Denmark; 2 Centre for Addiction and Mental Health, University of Toronto, Toronto, Canada; 3 Department of Biomedical Sciences, University of Copenhagen, Copenhagen, Denmark; 4 Aalborg Psychiatric Hospital, Aalborg University Hospital, Aalborg, Denmark; 5 Department of Clinical Medicine, Aarhus University, Aarhus, Denmark; 6 Mental Health Centre, Sct Hans, Capital Region of Denmark, Denmark; 7 Institute of Medical Genetics, Aarhus University, Aarhus, Denmark; 8 Mental Health Centre, Capital Region of Denmark, Denmark; 9 Center for Register Research, Institute of Economics, Aarhus University, Aarhus, Denmark; Johns Hopkins University, UNITED STATES

## Abstract

Mitochondria play a significant role in human diseases. However, disease associations with mitochondrial DNA (mtDNA) SNPs have proven difficult to replicate. An analysis of eight schizophrenia-associated mtDNA SNPs, in 23,743 Danes without a psychiatric diagnosis and 2,538 schizophrenia patients, revealed marked inter-allelic differences in mitochondrial haplogroup affiliation and nuclear ancestry. This bi-genomic dependence could entail population stratification. Only two mitochondrial SNPs, m.15043A and m.15218G, were significantly associated with schizophrenia. However, these associations disappeared when corrected for haplogroup affiliation and nuclear ancestry. The extensive bi-genomic dependence documented here is a major concern when interpreting historic, as well as designing future, mtDNA association studies.

## Introduction

Genetic variants in mitochondrial DNA (mtDNA)—and in nuclear genes coding for mitochondrial function—have been associated with disease [1–3]. More than 300 variants [4, 5] in mtDNA and genes involved in mitochondrial function [6] have been reported to cause mitochondrial disease, which is clinically characterized by complex metabolic, neurological, muscular and psychiatric symptoms [7, 8]. SNPs in mtDNA and mitochondrial haplogroups (mtDNA hgs), which are evolutionarily fixed SNP sets with a characteristic geographical distribution, have been proposed as potential disease modifiers[8]. This has been reported in neurological degenerative diseases such as Alzheimer’s disease [9–12] and Parkinson’s disease [12–14], metabolic diseases and cancers [15], as well as psychiatric diseases, notably schizophrenia (SZ) and bipolar disease [16–18].

Association studies of mtDNA variants and disease have been difficult to replicate[8]. However, the definition of a methodological paradigm for association studies with mtDNA variants [19] implicitly assumes that mtDNA variants are independent of the nuclear genome (gDNA). In a recent Danish study on mtDNA hgs and their nuclear ancestry, we demonstrated a marked difference in nuclear ancestry between individual mtDNA hgs [20]. This means that mtDNA hgs entail population stratification also at the level of gDNA. The effect of such a stratification on disease association will depend on the admixture structure of the particular population, the population history, epidemiology and genetic epidemiology of the disease, as well as the number of persons included in the study. The extensive fine-scale heterogeneity of gDNA and significant admixture documented in the UK [21] and Europe [22] further increase the risk of spurious false positive associations, if the mtDNA/gDNA interaction is not corrected in association studies.

Using DNA-array data from the Danish iPSYCH study on 2,538 SZ patients and 23,743 population controls, we show that eight mtDNA SNPs, previously associated with SZ [16–18, 23], exhibit considerable inter-allelic differences both with respect to mtDNA hg affiliation and nuclear ancestry. This phenomenon, which we name bi-genomic dependence, affects the association between an mtDNA SNP and mtDNA, as well as gDNA, and can lead to both false negative and positive associations with disease. We demonstrate that, in this cohort, it is only possible to replicate the association results for two of the original eight SNPs, when correcting for population stratification. Both mtDNA hg affiliation and nuclear ancestry affects the strength of association. Finally, we show that none of the SNPs are associated with SZ when examined on a particular mtDNA hg background, with correction for bi-genomic dependence. As none of previous studies of mtDNA SNPs have been performed with correction for population stratification, let alone bi-genomic dependence, our results indicate that all such published associations should be considered preliminary. In principle, this conclusion should not be limited to associations with SZ.

## Results

From a literature search of mtDNA SNPs previously associated with SZ, we identified eight that were also typed by the PsychChip, Table 1. PsychChip data from 23,743 normal Danes and 2,538 SZ patients (Detailed in S1 Table) showed that the SNPs were present in the population with frequencies varying from 0.2%– 20.6%, Table 1. There was no appreciable difference in mtDNA hg or nuclear ancestry distribution between controls and SZ patients, S1 and S2 Figs.

**Table 1:** Schizophrenia associated mtDNA SNPs, their frequency, mtDNA hg association, and link with disease.

Variant	Locus	Molecular effect	Effect on risk of SZ	% DK iPSYCH controls (SZ)	Theoretical mtDNA hg associations*
m.1438A [17]	MT-RNR1	n.a.	SZ↑	4.6 (4.8)	L0d, L1, C1b4, C4c2, M32′56, D5a2, I5c1, H1b1g, H2, H13a2b5, H14a2c, J1b1b1c, P2, P5
m.3197C [18]	MT-RNR2	n.a.	SZ↑	7.3 (8.2)	U5a′b, L3f1a1, H14b, U2e1a1a
m.3666A [16]	MT-ND1	p.G120G	SZ↑	0.3 (0.4)	L0d1c1a1a, L0d1b2b2c2, L1, M13c, H1ak, R9b2, F1a1c, U5a2b3a1
m.4769A [17]	MT-ND2	p.M100M	SZ↑	4.4 (4.6)	R2, B4a1a1c, B4a1a4, L0d2b1, H2a
m.9377G [16]	MT-CO3	p.W57W	SZ↓	0.5 (0.6)	G2a, Q2a1, D5b2, A2ac, H1c9a, U6a1b4, K1a4a1a2a, L2e, L3e2b1
m.10398G [23]	MT-ND3	p.T114A	SZ↑	20.6 (19.1)	K1, J, R11, B4m, B4c1c, R12′21, P4, U6a5c, K2a11, U8c, N1a1, W1e1a, W3a1d, N8, Y, S3, X2f1, R0a2k1, B5
m.15043A [18]	MT-CYB	p.G99G	SZ↑	5.7 (4.6)	M, N1a1, L2c2a, J1c2a3, T2f1a, U6a7, U2c1a
m.15218G [18]	MT-CYB	p.T158A	SZ↑	4.3 (5.2)	U5a1, M7a1a2, M10a1, HV1a′b′c, H13a2c

*Based on PhyloTree.

### Haplogroup distribution of mtDNA SNPs

**Fig 1:**
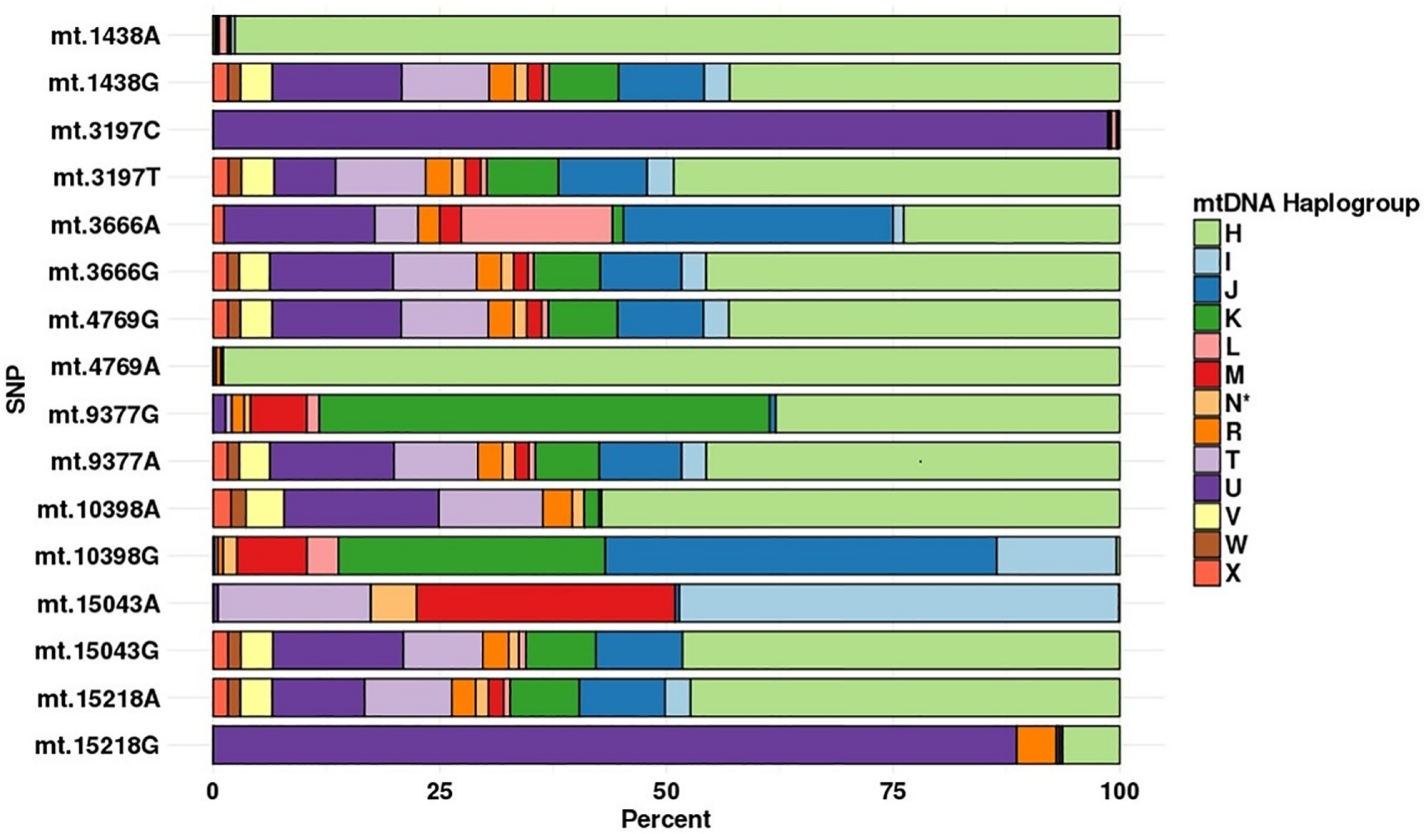
The percentage distribution of mtDNA hgs, per allele, among control individuals.

**Fig 2:**
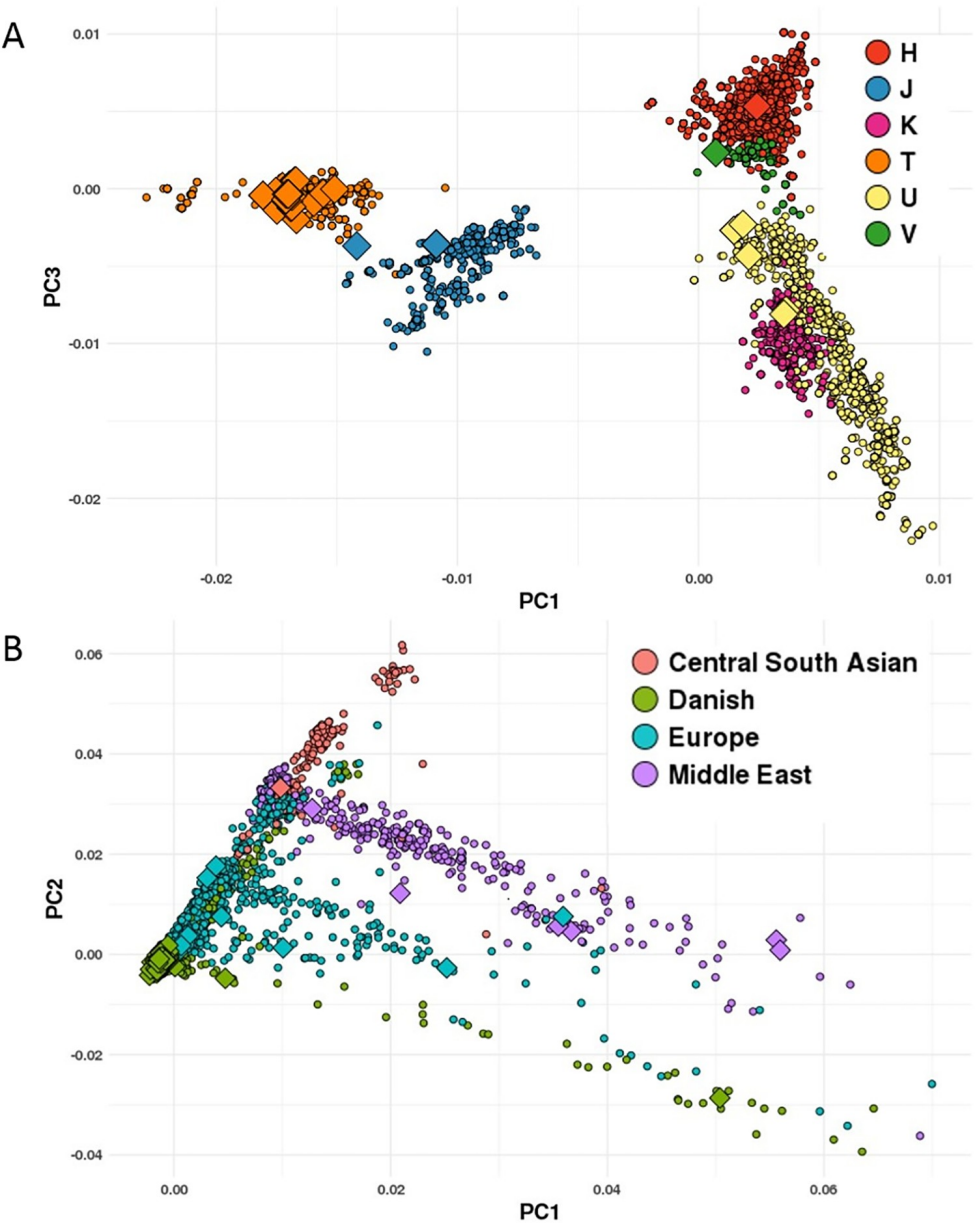
PCA of **A**. mtDNA (PC1 versus PC3) and **B**. gDNA (PC1 versus PC2), with the m.15043A (filled diamonds) and m.15043G (filled circles) alleles. Only persons with “European” mtDNA haplogroup (H, HV, V, U, K, J & T) were included. The PCA was performed using PCs defined by control samples.

The potential affiliation, based on PhyloTree, of SNPs to different mtDNA hgs is shown in Table 1, and the actual mtDNA hg distribution in the controls (not different from that of the SZ patients, S1 Fig) is shown in Fig 1. For all SNPs, there is a marked difference in the actual mtDNA hg distribution between the two alleles at the same position. Thus, when comparing persons with either of two alleles at the same mtDNA position, the comparison is between groups with widely differing mtDNA distributions. A PCA analysis of the mtDNA sequences in persons with either the A or G allele at position 15,043 is shown in Fig 2A. This analysis shows that the difference in mtDNA sequence, while subtle, indicates independent mtDNA distribution between the alleles.

### Nuclear ancestry of mtDNA SNPs

**Fig 3:**
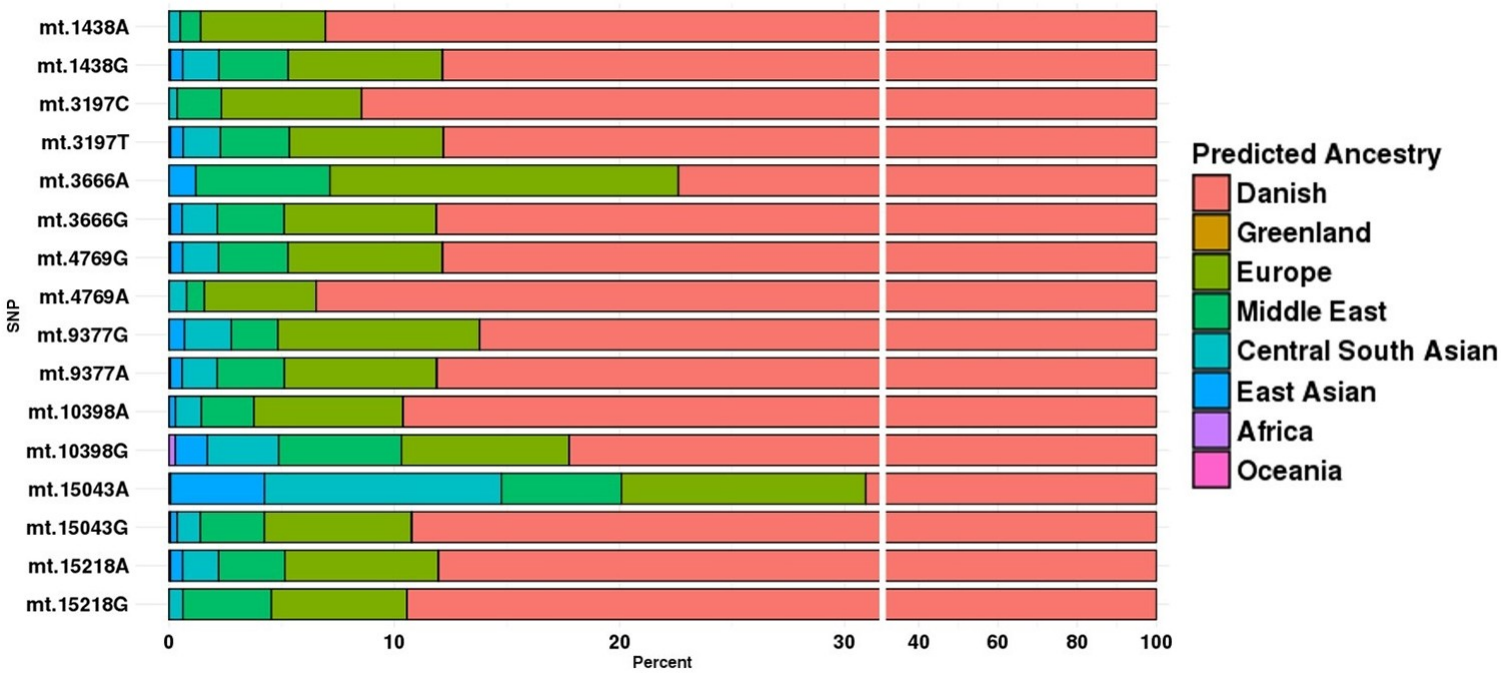
The percentage distribution of nuclear ancestries, per allele, among control individuals.

The distribution of nuclear ancestries in control persons as a function of each mtDNA allele revealed major differences, both between different positions (inter-SNP) in the mtDNA molecule and between different alleles (inter-allelic) at the same position, Fig 3. Apart from subtle differences in the distributions of Greenlandic and Asian ancestral affiliations, the distribution is similar in SZ patients, S2 Fig. The nuclear ancestry was statistically different between the alleles of seven of the eight SNPs assessed by chi-squared test; m.1438, m.3197, m.4769, m.10398 and m.15043 were statistically highly significant (P-value = 2.6e^-7^, 1.34e^-6^, 2.45e^-7^, 2.2e^-16^2.2e^-16^ respectively) and m.3666 and m15218 were statistically significant (P-value = 0.002 and 0.008 respectively whereas m.9377 was not statistically different. Most marked is the difference in Danish nuclear ancestry for m.15043 A/G, with an inter-allelic difference ~ 20 percent points in Danish ancestry, and the A-allele has a prominent Central South Asian ancestry not seen in the G-allele, Fig 3 and S2 Fig. PCA analyses of the gDNA, Fig 2B, of the two alleles, reveal subtle differences in the genomic structure independent of the nuclear ancestry between the two alleles. Thus, when comparing persons with either of two alleles at the same mtDNA position, there is a risk for comparison between groups with widely different ancestries, thus increased risk of confounding by population structure.

### Association between schizophrenia and mtDNA SNPs

**Fig 4:**
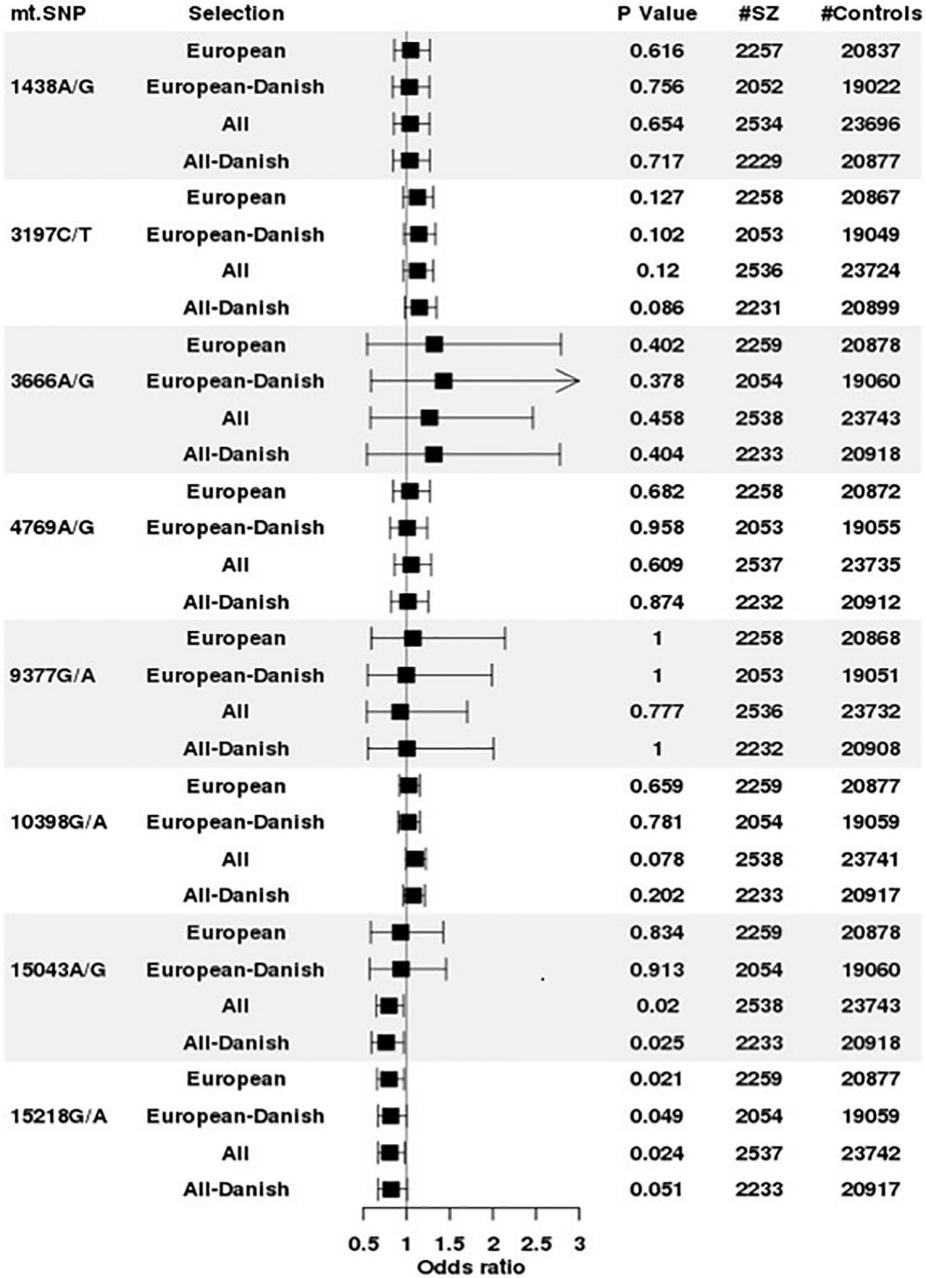
Association between individual mtDNA SNPs and schizophrenia as a function of the selection of the involved persons. (**All hgs**: All persons, **EU hgs**: persons with a “classical European” mtDNA hg; **EU_hgs_DANES**: persons with both a European mtDNA hg and a Danish nuclear ancestry and **ALL_hgs_DANES**: persons with a Danish nuclear ancestry).

The association of each mtDNA SNP with SZ was assessed (Fig 4). In consequence of the inter-allelic differences in mtDNA hg affiliations and nuclear ancestry demonstrated above, several association analyses were performed. Five SNPs, m.1438A, m.3197C, m.3666A, m.4769A, and m.9377G showed no association with SZ, both when all persons were included and where selection was made to reduce effects of varying mtDNA and nuclear ancestry affiliations. The m.10398G SNP was marginally significantly, while m.15043A was significantly associated with reduced risk of SZ in All and All-Danish mtDNA hgs. The m.15218G was associated significantly, or borderline significantly, with a reduced risk for SZ irrespective of the grouping, Fig 4. The high level of diversity in the PCA analysis of m.15043A (and the remaining SNPs—data not shown), Fig 2A, prompted us to examine whether a fixation of the mtDNA hg, i.e. limiting the analysis to persons with a specific hg, in cases where it was reasonably frequent, would result in significant associations. The result, Table 2, was the contrary—on a fixed mtDNA hg background, none of the SNPs exhibited a significant association with SZ.

**Table 2:** Association of SNPs with SZ in Danish nuclear ancestry and a specific mtDNA haplogroup.

mtDNA hg	Allele	Odds-ratio (95% cfi)	p	SZ (N)	Controls (N)
H	m.1438A	1.05 (0.84–1.29)	0.7	110	1,000
	m.3666A	1.12 (0.13–4.71)	0.7	2	17
	m.4769A	1.03 (0.83–1.28)	0.7	105	966
	m.9377A	0.95 (0.38–3.07)	0.8	1,046	9,917
	m.10398A	0.79 (0.18–7.14)	0.7	1,049	9,947
	m.15218A	1.09 (0.47–3.09)	1	1,045	9,900
J	m.3666A	0.74 (0.08–3.06)	1	2	22
K	m.9377A	0.90 (0.38–2.58)	0.8	133	1,509
	m.10398A	1.40 (0.90–2.12)	0.1	33	106
U	m.3197C	1.08 (0.85–1.38)	0.6	189	1,572
	m.3666A	2.59 (0.46–10.13)	0.2	3	10
	m.15218A	0.81 (0.63–1.04)	0.1	211	1,939
T	m.15043A	0.99 (0.59–1.58)	1	22	208

## Discussion

Here we show the dependence of mtDNA SNPs with both mtDNA hgs and gDNA clusters due to population structure and shared demographic history of mtDNA and gDNA, we have called this relationship “bi-genomic dependence”. This has the consequence that an association between a particular allele, in a specific SNP, and disease is not exclusively the result of the presence of that particular allele; since each mtDNA allele is associated with the unique distributions of both mtDNA hgs and gDNA clusters. Furthermore, bi-genomic dependence can be accounted for by including mtDNA and gDNA principle components from PCAs in association analyses—thus incorporating a bi-genomic measure of population stratification and admixture into mtDNA association analyses. Currently, such a measure of bi-genomic dependence is not incorporated into studies of mtDNA-disease-association which consider an association as evidence for a specific effect of an allele on the function of a protein or RNA coded for by the mtDNA and, consequently, as a cause of pathophysiological changes.

The linkage disequilibrium between different alleles of a SNP and mtDNA hgs and sub-hgs is not surprising as hgs are defined by series of evolutionarily conserved SNPs. The particular distribution of subsets of mtDNA hgs, sub-hgs and individual SNPs that are associated with a particular allele at a specific SNP will depend on the population history and the extent and source of admixture. In most countries, and in particular in Europe, such history is very complicated and incompletely clarified. m.10398G was found associated with SZ in Han Chinese, however, when the cohort was broken down with respect to mtDNA hgs, the association disappeared [24]. This illustrates that a specific allele’s mtDNA hg distribution may induce spurious association with SZ. Spurious associations between mtDNA SNPs and a particular phenotype, when restricted to persons of a specific mtDNA hg [25], may however be due to population stratification at the sub-hg level. In the iPSYCH cohort there was no association between SZ and any of the European mtDNA hgs (Data not shown).s mtDNA replicates independently of the cell cycle and without recombination, mtDNA hgs and SNPs should be independent of the gDNA, but only if the population is infinitely large and in the absence of population substructure. This is often not the case, due to geographical population substructure, recent admixture, socially and culturally defined restrictions in choice of spouse. A recent study showed that most Danish grandparents to present-day high school students chose spouses within a short distance of their birthplace [26]. This practice will, with time, lead to regionalization, and a southwestern to northeastern gradient was found [26]. Furthermore, immigrants may seek a partner from within their ethnic community. Such effects have been eliminated in some studies by restricting the participants in mtDNA association studies to persons with a three-generation presence in the population. However, it has not been documented that this is sufficient to obviate association or linkage disequilibrium between mtDNA SNPs and specific gDNA clusters. Extensive gDNA micro-scale heterogeneity has been documented in the UK [21] and Western France [27] and admixture has been an important factor in the accretion of the present-day genomic variation of Europe [22, 28]. The UK study [21] showed that this is not just a result of recent demic changes; however, recent migrations may lead to widespread bi-genomic dependence.

Schizophrenia is a complex syndromic disease [29] with geographically varying prevalence[30] and characterized by a markedly elevated prevalence among first and second generation immigrants [31, 32], particularly among persons with dark skin moving to Nordic latitudes [33]. These epidemiological characteristics of SZ obviously increase the risk of spurious associations caused by subtle admixture and bi-genomic dependence. However, it does not *per se* refute the mitochondrial pathogenic paradigm [34] where variation in mitochondrial function, believed to interfere with ATP production [35, 36], inflammation and signaling [37, 38] as well as Ca^2+^-homeostasis [39], and apoptosis [38], is considered to be of paramount importance for development of disease. Several neuroanatomical post-mortem findings in SZ brains indicate perturbed mitochondrial function [40], but such findings are difficult to distinguish from changes caused by drug treatment.

The iPSYCH data are prospective and signs of immigration are apparent [20], but they also showed that the variation in ancestry differed greatly between mtDNA hgs—even within traditional European hgs, i.e. mtDNA hg U, where ancient European sub-hgs occurred together with U-sub-hgs of recent Near Eastern and Central Asian origin [20]. Thus, bi-genomic dependence is likely to be a confounder and may lead both to false positive as well as false negative associations with disease. The method of correction for bi-genomic dependence in association studies will depend on the specific mtDNA SNP examined, the population structure and history, as well as the size of the study population.

If population stratification involving gDNA is inherent when performing association studies with mtDNA SNPs, it should be expected that diseases with geographically varying prevalence would be likely to find associated with specific mtDNA SNPs. The largest mtDNA association study to date [18] found mtDNA SNPs associated with ulcerative colitis, exhibiting a European North-South and East-West gradient [41], and with multiple sclerosis exhibiting a longitudinal prevalence gradient [42] and effect of immigration [43]. The same study found that the prevalence of mtDNA SNPs associated with Parkinson’s disease was lower in African and Asian people [44]. Furthermore, the incidence of primary biliary cirrhosis is very high in North East England, 50% lower in the rest of England and Scandinavia, and 90% lower in the Middle East and Asia [45].

A major problem with the interpretation of mtDNA SNP variants is the difficulties associated with performing a meaningful and reproducible assessment of mitochondrial function. In vitro studies of mitochondrial function, e.g. enzymatic activity measurements of OXPHOS components in cells, tissues [46] or cybrids [47] as well as allotopic expression [15], are difficult to interpret as they also interfere with the inherent cellular control of mitochondrial function [38]. Furthermore, it should be noted, that mtDNA hgs and sub-hgs are cladistics groups and not functional units. Thus, in the Danish population, the U-hg is composed of a range of sub-hgs, e.g. U5a, U5b, U6, U7, and U8, with widely differing nuclear ancestries, reflecting migrations rather than selection [20]. It is thus meaningless to ascribe a specific functional effect to a particular mtDNA hg—without having carefully examined both mtDNA and nuclear genetic variation and corrected for stratifications in both.

Previous conflicting studies of disease associations with mtDNA have been suggested to be the result of insufficient power [48], insufficient stratification respect to sex, age, geographical background [49] or population admixture [50], or the use of small areas of recruitment risking “occult” founder effects [51]. The fact that careful control, as here, of these factors and the bi-genomic dependence, results in none of eight previously SZ associated mtDNA SNPs being associated with SZ in the very large Danish iPSYCH cohort, suggests that previously reported associations could indeed be spurious findings due to cryptic population stratification. Meta-analyses pooling studies from different populations [15] does not necessarily solve this problem—it may aggravate it by introducing further sub-stratification of the total population analyzed. The extensive bi-genomic dependence demonstrated in the Danish population makes this phenomenon the most parsimonious explanation of non-replicable associations with mtDNA variants, not only associations with SZ, but obviously bi-genomic dependence can interfere with associations between mtDNA and all types of diseases and traits.

## Materials and methods

### Ethics statement

The iPSYCH cohort study (www.ipsych.au.dk) is register-based using data from Danish national health registries. The study was approved by the Scientific Ethics Committees of the Central Denmark Region (www.komite.rm.dk) (J.nr.: 1-10-72-287-12) and executed according to guidelines from the Danish Data Protection Agency (www.datatilsynet.dk) (J.nr.: 2012-41-0110). Passive, but not informed, consent was obtained, in accordance with Danish Law nr. 593 of June 14, 2011, para 10, on the scientific ethics administration of projects within health research.

### SZ patients and controls

As part of the iPSYCH recruitment protocol, 23,743 controls, born between May 1^st^ 1981 and Dec 31^st^ 2005 were selected at random from the Danish Central Person Registry. Among persons born within the same time span 2,538 persons assigned an ICD-10 F20 were identified in the Danish National Patient Registry. All were singletons, were alive one year after their birth, and had a mother registered in the Danish Central Person Registry. DNA was available from DBS cards obtained from the Danish Neonatal Screening Biobank at Statens Serum Institut [52] Demographics of patients and controls are given in S1 Table.

### Genetic analysis and mtDNA SNPing

From each DBS card two 3.2-mm disks were excised from which DNA extracted using Extract-N-Amp Blood PCR Kit (Sigma-Aldrich, St Louis, MO, USA)(extraction volume: 200 μL). The extracted DNA samples were whole genome amplified (WGA) in triplicate using the REPLIg kit (Qiagen, Hilden, Germany), then pooled into a single aliquot. Finally, WGA DNA concentrations were estimated using the Quant-IT Picogreen dsDNA kit (Invitrogen, Carlsbad, CA, USA). The amplified samples were genotyped at the Broad Institute (MA, USA) using the PsychChip (Illumina, CA, USA) typing 588,454 variants, developed by the Psychiatric Genetic Consortia. We then isolated the 418 mitochondrial loci and reviewed the genotype calls, before exporting into the PED/MAP format using GenomeStudio (Illumina, CA, USA). Haplo-grouping of mtDNA was performed using the defining SNPs reported in www.phylotree.org [53].

### Nuclear ancestry

Nuclear ancestry estimation was done using ADMIXTURE 1.3.050 in the supervised approach. Briefly, reference populations consisting of Human Genome Diversity Project (HGDP) (http://www.hagsc.org/hgdp/), a Danish (716 individuals) and a Greenlandian (592 individuals) genotyping SNP data set were used. The final reference data set consisted of 103,268 autosomal SNPs and 2,248 individuals assigned to one of nine population groups: Africa, America, Central South Asia, Denmark, East Asia, non-Danish Europe, Greenland, Middle East and Oceania. The number of clusters, K was set to eight, based on principal component analysis clustering (data not shown). The subpopulations were merged with the reference population data set and analyzed using ADMIXTURE. For prediction of the ancestry of individuals within the mtDNA hgs we created a random forest model [54] based on the reference data set, with the clusters Q1-8 as predictors and population groups as outcome. Thus, the ancestry analysis of the individual person was the result of a supervised prediction. Prediction was done using R3 version 3.2.2, using the Caret package.

### Statistics

The statistical significance of differences in mtDNA SNP proportions between controls and SZ patients was assessed using a permutation version of Fisher’s exact test. Samples with missing sequence data were excluded. To assess differences in allele distribution within the predicted ancestries we used Pearson’s Chi-squared test. Ancestries with allele counts below six were not included. Calculations were performed using R (version 3.1.3). Principal component analysis (PCA), was prepared using PLINK (v.1.90b3.31). For the PCA the reference population variants were extracted from the iPSYCH control sample, LD pruned (indep-pairwise 50 5 0.5) and allowing only SNPs with 99% genotyping rate. Prior to PCA of mtDNA data, samples were loaded into GenomeStudio (version 2011.a), a custom cluster was created using Gentrain (version 2), following automatic clustering, all positions with heterozygotes were manually curated. The data was exported relative to the forward strand using PLINK Input Report Plug-in (version 2.1.3). Eigenvectors were calculated using PLINK (v1.90b3.31). PCA plots were created using the package ggplot2 (version 1.0.1) in R (version 3.1.3).

## Supporting information

S1 Table Demographics and nuclear ancestry and mtDNA haplogroups of the cohort. (DOCX)

S1 Fig Distribution of mtDNA haplogroups of schizophrenic patients with specific SNPs. (TIF)

S2 Fig Distribution of nuclear ancestries of schizophrenic persons with specific SNPs. (TIF)
