# Supplementary material for: Schizophrenia-associated mt-DNA SNPs exhibit highly variable haplogroup affiliation and nuclear ancestry: Bi-genomic dependence raises major concerns for link to disease
Source: PLoS One. 2018 Dec 10;13(12):e0208828. doi: 10.1371/journal.pone.0208828 (PMC6287820; doi:10.1371/journal.pone.0208828)
Supplement: S1 Table — (DOCX) [file pone.0208828.s001.docx]

**S1 Table.** Demographics and GGA and mtDNA haplogroups of the cohort

| **Characteristics** | **Controls (n = 23,743)** | **Schizophrenia (n = 2,538)** |
| --- | --- | --- |
| **Age (Median and range), years** | 23 (12 – 36) | 28 (17 – 36) |
| **Sex (Male/Female)** | 12,067 / 11,676 | 1,469 / 1,069 |
| **Genogeographic affinity:** |  |  |
| Danish | 20,918 (88.10 %) | 2,233 (87.98 %) |
| European | 1,584 (6.67 %) | 195 (7.68 %) |
| Middle Eastern | 724 (3.05 %) | 59 (2.32 %) |
| Central South Asian | 365 (1.54 %) | 44 (1.73 %) |
| African | 15 (0.06 %) | 1 (0.04 %) |
| Greenlandian | 4 (0.02 %) | 3 (0.12 %) |
| **mtDNA haplogroup:** |  |  |
| H | 10,817 (45.6 %) | 1,153 (45.4 %) |
| U | 3,205 (13.5 %) | 375 (14.8 %) |
| T | 2,184 (9.2 %) | 231 (9.1 %) |
| J | 2,123 (8.9 %) | 255 (10.0 %) |
| K | 1,753 (7.4 %) | 164 (6.5 %) |
| V | 796 (3.4 %) | 81 (3.2 %) |
| I | 655 (2.8 %) | 55 (2.2 %) |
| R | 646 (2.7 %) | 65 (2.6 %) |
| X | 390 (1.6 %) | 25 (1.0 %) |
| M | 386 (1.6 %) | 31 (1.2 %) |
| W | 319 (1.3 %) | 28 (1.1 %) |
| N | 296 (1.2 %) | 62 (2.4 %) |
| L | 173 (0.7 %) | 13 (0.5 %) |
