# Supplementary figures and images for: Schizophrenia-associated mt-DNA SNPs exhibit highly variable haplogroup affiliation and nuclear ancestry: Bi-genomic dependence raises major concerns for link to disease
Source: PLoS One. 2018 Dec 10;13(12):e0208828. doi: 10.1371/journal.pone.0208828 (PMC6287820; doi:10.1371/journal.pone.0208828)

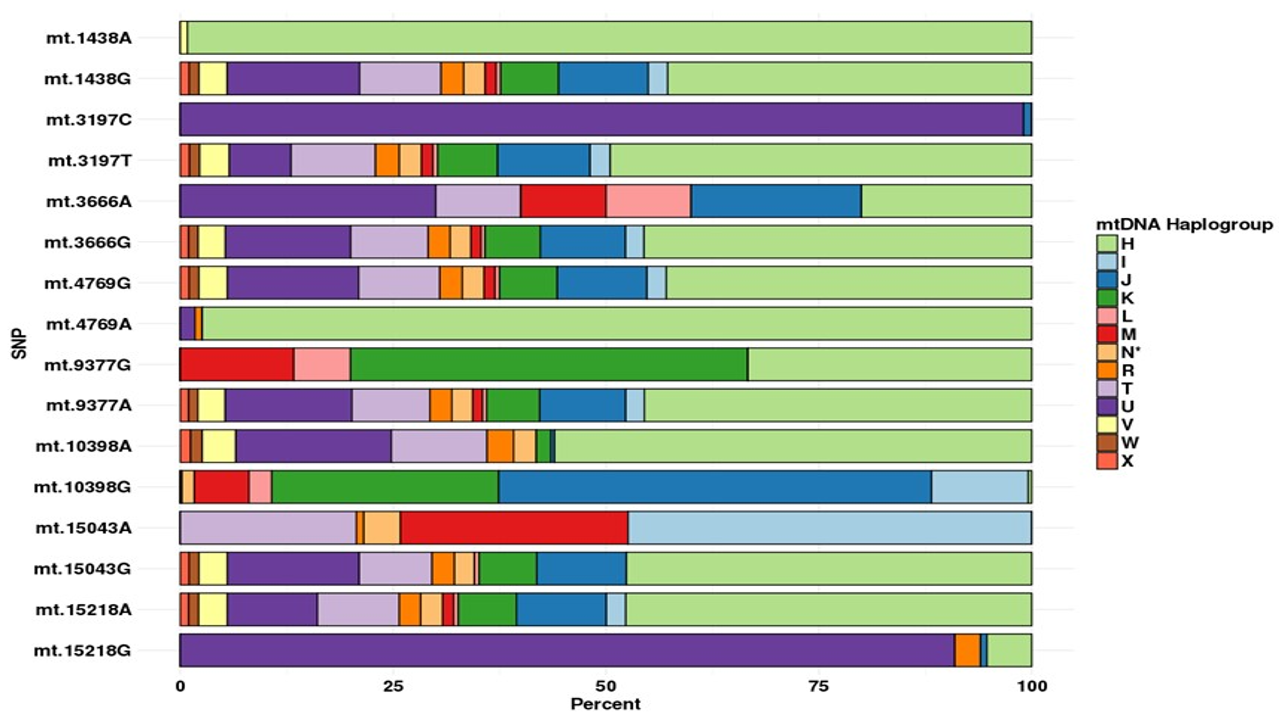

Supplement: S1 Fig — (TIF) [file pone.0208828.s002.tif]

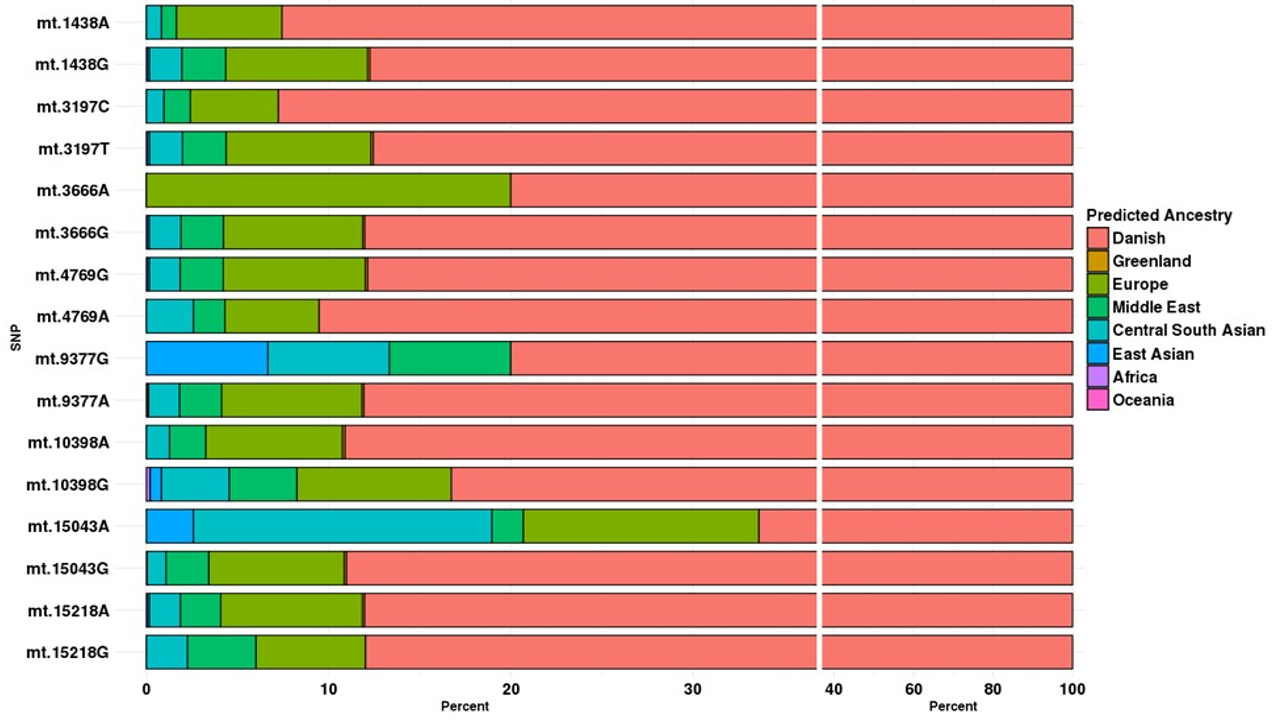

Supplement: S2 Fig — (TIF) [file pone.0208828.s003.tif]
